# 

**Published:** 1890-09

**Authors:** 


### Communications.

### AMERICAN ACADEMY OF DENTAL SCIENCE.

*Editor of the American Journal of Dental Science:*

The Twenty Third annual meeting of the American Academy of Dental Science will be held in Boston, on Wednesday, November 12, 1890.

The annual address will be delivered by W. W. H. Thackston, M.D., D.D.S., of Farmville, Va.

EDWARD N. HARRIS, D.D.S.,
Cor. Secretary,
2 Park Square, Boston, Mass.

---

### THE DENTAL PROTECTIVE ASSOCIATION.

*Editor of the American Journal of Dental Science:*

At a meeting of the Michigan Dental Association, the principal points in Dr. Crouse's remarks were:

First, that by continuing in some such an organization as the Dental Protective Association, we band the strength of ten thousand men into one. All defence necessary, can be made with but little more expense than would be required for an individual. The preparation of one case will answer for all, all evidence can be collected better by an association than in any other way.

Second, that it is very important that all get into the association at this time, for the reason, that an appealed suit to the Supreme Court before the formation of the association, will probably be decided in favor of the Crown Company, owing to the deficiency in evidence and imperfect presentation. If the Crown Company win this suit they will send notices all over the United States, thereby demoralizing the profession and causing many to pay them money, who should be in the protective association, and thereby avoid that calamity.

The Protective Association will be ready with new evidence and a new record and can take care of its members against any claims of the Crown Company.

If you are not in the association the association will not take care of you when the time comes. If each man in the profession will pay $10 and assume a responsibility of ten more, without any further assessments, we will have an organization that is sure to break up all this abuse and save the dental profession an annual outlay, of, on an average of $100 each, to unjust claimants.

Remember it will cost more than $10 later to join, and will certainly cost more than that if you do not protect yourselves.

WM. CLELAND,
Sec'y Mich. Dental Asso'n.

*Resolved*, That the Michigan Dental Association, heartily approves the aim and plan of the Dental Protective Association and that it is further

*Resolved*, That it is the duty of every member of the dental profession in this State to join the Dental Protective Association.

*It is also Resolved*, That Dr. Crouse be requested to furnish the dental journals with an abstract of his remarks for publication.

W. H. DORRANCE,
W. D. SAUNDERS,
—— DOUGLASS,
Committee.

### To Readers and Correspondents.

Communications intended for publication, and exchange journals and papers, should be addressed to the Editor of The American Journal of Dental Science, No. 845 N. Eutaw St. Baltimore, Md. All letters relating to the business of the Journal, or containing cash remittances, to be directed to Messrs. Snowden & Cowman, No. 9 W. Fayette Street, Baltimore, Md. Articles for publication should be received by the Editor at least two weeks previous to the first of the month on which the number in which it is designed for them to appear, is issued.

☞ The American Journal of Dental Science will contain fifty pages of reading matter in each number, making a volume of about Six Hundred pages,

EXCLUSIVE OF ADVERTISEMENTS.

